# Deformed wing virus prevalence and load in honeybees in South Africa

**DOI:** 10.1007/s00705-020-04863-5

**Published:** 2020-11-02

**Authors:** Flaviane S. de Souza, Michael H. Allsopp, Stephen J. Martin

**Affiliations:** 1 School of Environment and Life Sciences, The University of Salford, Manchester, M5 4WT UK; 2 Universidade Federal do Recôncavo da Bahia, Rua Rui Barbosa 710, Cruz das Almas, Bahia, 44380-000 Brazil; 3 ARC-Plant Protection Research Institute, P/Bag X5017, Stellenbosch, 7599 South Africa

## Abstract

Deformed wing virus (DWV) is an emerging honeybee pathogen that has appeared across the globe in the past 40 years. When transmitted by the parasitic varroa mite, it has been associated with the collapse of millions of colonies throughout the Northern Hemisphere. However, despite the presence of the mite in the Southern Hemisphere, infested colonies survive. This study investigated the prevalence of DWV genotypes A, B and C along with their viral loads in South Africa and compared the findings with recent data from Brazil, the UK and the USA. We found that DWV-B was the most prevalent genotype throughout South Africa, although the total DWV viral load was significantly lower (2.8E+07) than found in the Northern Hemisphere (2.8E+07 vs. 2.7E+10, *p* > 0.00001) and not significantly different to that found in Brazil (5E+06, *p* = 0.13). The differences in viral load can be explained by the mite resistance in Brazil and South Africa, since mite-infested cells containing high viral loads are removed by the bees, thus lowering the colony's viral burden. This behaviour is much less developed in the vast majority of honeybees in the Northern Hemisphere.

Deformed wing virus (DWV) is a newly emerging pathogen that within 40 years from its discovery has become the most widespread insect virus. DWV has been detected in over 50% of all honeybee colonies in 32 countries at high viral loads and in 65 arthropod species spanning eight orders [1]. Prior to the global spread of a highly pathogenic parasitic mite, *Varroa destructor*, there was a least one known honey bee colony collapse linked to high DWV loads (>10^7^ viral particles/bee) (unpublished data, SJM). Recently, more-sensitive testing has shown that the DWV prevalence in varroa-free honeybee populations is highly variable, being absent in Australia [2], low in Hawaii [3], and high in Newfoundland [4], but viral loads are typically very low. The recent success of DWV is due to its close association with an ectoparasitic mite (*Varroa destructor*) that lives on honeybees and during the past 70 years has spread globally [5]. Molecular evolutionary clocks [6] have demonstrated the possible appearance of widespread DWV-related pathologies with the arrival of Varroa mites. The mite has introduced a new viral transmission route when feeding on the honeybee, causing elevated DWV levels and selecting for highly virulent DWV variants [4] or the appearance of pathogenic strains that outcompete other DWV variants [7]. It is these virulent variants that contribute to the death of the colony. DWV consists of three master-variants (DWV-A, -B, and -C) and various recombinants, each with a different prevalence [8]. In North America and the UK, DWV-A was initially the dominant genotype, but over the past 20 years, DWV-B prevalence has increased in the USA [9] or has almost replaced the DWV-A variant in the UK [10]. DWV-C remains a rare variant. However, little is known about the distribution of DWV variants outside these two countries, especially those in the Southern Hemisphere, where natural resistance to the varroa mite is frequency found [11].

Therefore, the aim of this study was to determine the viral load and prevalence of the three DWV master-variants (A, B and C) in South Africa. South Africa has two honeybee subspecies, the Cape honeybee (*Apis mellifera capensis*), which is found in the southern fynbos ecosystem, and the Savanna honeybee (*A. m. scutellata*), which is found throughout the rest of the country (Fig. 1a). Both subspecies are resistant to many of the pathogens and parasites that plague honeybee populations in the Northern Hemisphere and thus require limited pest management [12]. Most importantly, after the arrival of the varroa mite in 1997 to the Cape Region of South Africa [13], mite resistance appeared after 3-5 years and 6-7 years in Cape and Savanna honeybees, respectively [14]. Globally, the natural evolution of mite resistance in *A. mellifera* honeybees is an uncommon trait, since biannual or annual acaricides treatments are required to ensure a colony's survival. Thus, we used honeybee samples of both subspecies collected across the south of the country to compare DWV variant distribution and viral load.

**Fig. 1:**
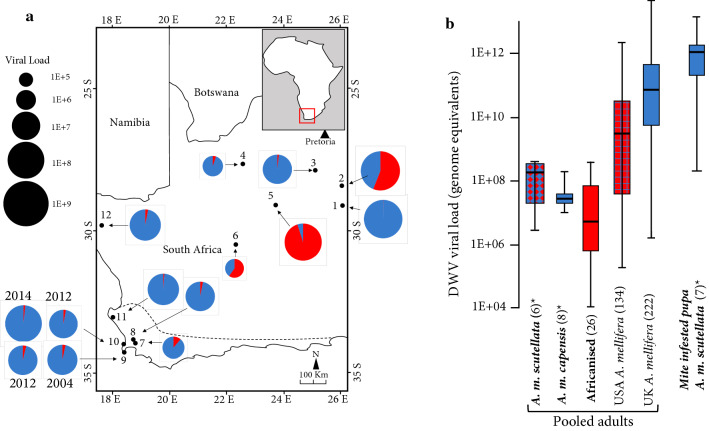
**a** DWV load and prevalence (DWV-A, red; DWV-B, blue) throughout South Africa. The area south of the dotted line is occupied by *A. m. capensis,* the area north of the line is occupied by *A. m. scutellata*, and there is a hybrid buffer contact zone that expends 300 km north of the dotted line. Sampling locations are as follows: 1, Bloemfontein; 2, Theunissen; 3, Christiana; 4,Tswalu Kalahari; 5, Douglas; 6, Three Sisters; 7, Le Verger; 8, Drie Koppen; 9, Cape Point; 10, Robben Island; 11,- Langebaan; 12, Nababeep. **b** Comparison of the DWV viral load and genotype in this study (*) with these found in several other countries. Mite-resistant populations are in bold, and the number of colonies sampled is in parentheses. Where neither DWV-A (red) nor DWV-B (blue) dominates, the most prevalent variant is indicated by the base colour of the hatched pattern

Between 2007 and 2018, 20 colonies were sampled from 12 locations across South Africa (Table 1; Fig. 1a). Each sample consisted of several hundred adult bees collected from brood frames and stored at  – 20 °C. In addition, a single sample of adult bees was collected in 2004 from Cape Point. In 2018, seven *A. m. scutellata* mite-infested pupae were sampled from two locations (Douglas and Three Sisters), since this gives us the best chance of confirming the presence or absence of DWV. Pools of 10 adult bees or one infested pupa from each colony were analysed using an assay developed recently by Kevill et al. [15]. Briefly, RNA was extracted from a 30-mg sub-sample of the pool bees or single pupa, which were ground to a power using a pestle and mortar in liquid nitrogen. After the RNA was quantified using a NanoDrop spectrophotometer, each sample was diluted to 50 ng/µl. RT-PCR was performed using a SensiFAST SYBR No-Rox One-Step Kit with primers specific designed to recognize the RdRp region of the DWV genomic RNA to separate out DWV-A, DWV-B, and DWV-C variants of the RdRp gene. A small number of DWV-A and DWV-B samples were sequenced to confirm their identity. Viral load was expressed as genome equivalents per bee, using the following equations [15]:

1. copy number RNA = (concentration RNA (ng/L) × 6.022 × 10^23^ )/ (fragment length base pairs × 109 × 325)
2. genome equivalents = (average copy number RNA) × (RNA dilution factor) × (elution volume of RNA) × (proportion of bee material)

**Table 1:** Collection and viral data for each colony sample of *A. m. scutellata* and *A. m. capensis* adults or *A. m. scutellata* mite-infested pupae

Bee race	Location	Location code (Fig. 1a)	Year collected	Genome equivalents DWV-A	Genome equivalents DWV-B	Genome equivalents DWV-C
	**Pooled adult workers**					
*A. m. scutellata*	Bloemfontein	1	2017	5.55E+05	3.05E+08	0.00E+00
*A. m. scutellata*	Theunissen	2	2017	2.41E+08	1.89E+08	0.00E+00
*A. m. scutellata*	Christiana	3	2017	9.23E+05	5.35E+07	0.00E+00
*A. m. scutellata*	Tswalu Kalahari	4	2007	4.62E+05	8.46E+06	0.00E+00
*A. m. scutellata*	Douglas	5	2008	3.33E+08	1.73E+07	0.00E+00
*A. m. scutellata*	Three Sisters	6	2017	1.57E+06	1.06E+06	0.00E+00
*A. m. capensis*	Le Verger	7	2008	7.28E+05	2.40E+07	0.00E+00
*A. m. capensis*	Drie Koppen	8	2017	1.11E+06	8.83E+06	0.00E+00
*A. m. capensis*	Cape Point	9	2004	1.05E+06	2.95E+07	0.00E+00
*A. m. capensis*	Cape Point2	9	2012	9.82E+05	2.53E+07	0.00E+00
*A. m. capensis*	Robben Island	10	2012	1.65E+06	5.58E+07	0.00E+00
*A. m. capensis*	Robben Island 2	10	2014	3.26E+06	2.03E+08	0.00E+00
*A. m. capensis*	Langebaan	11	2017	1.99E+05	1.84E+07	0.00E+00
*A. m. cape/scut hybrid*	Nababeep	12	2008	6.22E+05	2.08E+07	0.00E+00
	**Mite-Infested pupae**					
*A. m. scutellata*	Douglas/Stellenbosch (1) *	5	2018	2.66E+06	2.04E+08	4.19E+07
*A. m. scutellata*	Douglas/Stellenbosch (2) *	5	2018	7.94E+06	2.43E+12	2.33E+09
*A. m. scutellata*	Douglas/Stellenbosch (3) *	5	2018	6.10E+06	4.02E+12	2.04E+08
*A. m. scutellata*	Douglas/Stellenbosch (4) *	5	2018	1.57E+06	1.30E+09	0.00E+00
*A. m. scutellata*	Three Sisters (1)	6	2018	3.48E+06	5.64E+11	2.14E+08
*A. m. scutellata*	Three Sisters (2)	6	2018	1.03E+06	1.04E+12	7.02E+08
*A. m. scutellata*	Three Sisters (3)	6	2018	2.42E+06	2.32E+12	7.63E+08

*Colonies moved from Douglas in 2008 to the Stellenbosch area

All statistical comparisons of viral load were conducted using the Mann–Whitney U test due to the non-normal data distribution, and median load values are presented throughout.

We found that, throughout South Africa, the DWV-B variant is the prevalent genotype in adult bees especially in *A. m. capensis*, with DWV-A being more prevalent in just three locations, all occupied by *A. m. scutellata* (Table 1; Fig. 1a). Furthermore, the viral loads in adults of South African honeybees were significantly lower (U = 702, *p* < 0.00001) lower than the viral loads of DWV in the Northern Hemisphere, i.e., UK and USA, but not significantly different (U = 112, *p* = 0.13) to that found in Brazil [16], where the honeybee population is also resistant to *V. destructor*. The same analytical pipeline [15] was followed in all these studies. The viral levels in the seven *A. m. scutellata* mite-infested pupae sampled in this study (Table 1) were significantly higher (1E+12 vs. 2E+08, U = 0, *p* = 0.0034) than present in adults, as expected [17]; however, they were dominated by the DWV-B genotype, whereas previous collected adults contained mainly DWV-A. Again, this is not uncommon, since viral genotypes in a location are variable over time [10]. Furthermore, DWV-A/B recombinants may exist but would not be detected using this method. The infested pupae were the only samples in which DWV-C was detected (Table 1), and its rarity is typical of this variant in adult honeybees studied in the USA, the UK [10] and Brazil [16].

The only previous viral survey of *A. m. scutellata,* mainly in the Pretoria area, failed to detect DWV-A in any of the samples, but DWV-B was detected in four apiaries [18], supporting the prevalence of DWV-B in South Africa. A colony with high loads of DWV (detected using ELISA) was reported in South Africa prior to the arrival of varroa mites in 1997 [19], indicating that DWV was circulating in South Africa prior to the arrival of the varroa mites.

A comparable study was conducted across Brazil [16], where Africanised bees are common. These are a man-made hybrid between *A. m. scutellata* from South Africa and honeybees from Europe. Africanised bees are also resistant to varroa mites, and despite the mite’s presence since the 1970s and 1980s, Brazil remains unique in that DWV-A remains the dominant genotype throughout the country, with DWV-B dominating in only one of 26 colonies studied [16]. This is unexpected, since under both controlled [20, 21] and natural field conditions [9, 10], DWV-B outcompetes and replaces DWV-A. This competition explains why there is no significant difference (χ^2^ = 3.3, *p* = 0.07) between the number of DWV-A- or DWV-B-dominant colonies between the UK [10] and South Africa, which have both been infested with varroa mites for over 30 years. Similarly, in the USA in 2010, DWV-B dominated just 3% of colonies, but this figure rose to 66% in 2016 [9], indicating the speed of change.

While the honeybees in Brazil and South Africa are both resistant to varroa mites, they have different DWV genotypes. However, in both countries, the viral load is significantly lower than in the UK or the USA (Fig. 1b). This may be explained by the finding that, in both Brazil and South Africa, the adult workers detect and remove a greater amount of infested brood [22] than is the case in the Northern Hemisphere, thereby reducing the viral burden on the colony. Although the highest viral loads were detected in mite-infested pupae, over 50% are removed by the bees prior to maturity [22], and the remaining infested pupae have reduced longevity as adults. Both of these factors greatly reduce the viral load in adult bees, as was seen in South Africa and Brazil, but not in the UK or the USA (Fig. 1b), where the rate of removal of mite-infested pupae is low.

Some caution is required in interpreting these results, since the data are derived from a small number of colonies sampled over an 11-year period. Now, a more systematic approach [see 9, 10] across South Africa and beyond is needed to understand the wider impact of DWV on African honeybees. However, based on the current data, it may be the lower viral loads found in South Africa and Brazil rather than the variant (DWV-A vs DWV-B) that reduces the burden of DWV on the honeybees.
